# Can supplementary pollen feeding reduce varroa mite and virus levels and improve honey bee colony survival?

**DOI:** 10.1007/s10493-020-00562-7

**Published:** 2020-10-30

**Authors:** Gloria DeGrandi-Hoffman, Vanessa Corby-Harris, Yanping Chen, Henry Graham, Mona Chambers, Emily Watkins deJong, Nicholas Ziolkowski, Yun Kang, Stephanie Gage, Megan Deeter, Michael Simone-Finstrom, Lilia de Guzman

**Affiliations:** 1 USDA-ARS, Tucson, AZ USA; 2 USDA-ARS, Beltsville, MD USA; 3 Arizona State University, Tempe, AZ USA; 4 University of Arizona, Tucson, AZ USA; 5 USDA-ARS, Baton Rouge, LA USA

**Keywords:** Overwinter survival, Varroa migration, Tolerance, Resistance

## Abstract

*Varroa destructor* is an ectoparasitic mite of immature and adult honey bees that can transmit several single-stranded RNA viruses to its host. Varroa reproduce in brood cells, and mite populations increase as colonies produce brood in spring and summer. Mite numbers also can sharply rise, particularly in the fall, by the migration of varroa into hives on foragers. Colonies with high levels of varroa and viruses often die over the winter. Feeding colonies pollen might keep virus levels low and improve survival because of the positive effects of pollen on immunity and colony growth. We compared varroa and virus levels and overwinter survival in colonies with (fed) and without (unfed) supplemental pollen. We also measured the frequency of capturing foragers with mites (FWM) at colony entrances to determine its relationship to varroa and virus levels. Colonies fed supplemental pollen were larger than unfed colonies and survived longer. Varroa populations and levels of Deformed wing virus (DWV) rose throughout the season, and were similar between fed and unfed colonies. The growth of varroa populations was correlated with FWM in fed and unfed colonies, and significantly affected DWV levels. Increasing frequencies of FWM and the effects on varroa populations might reduce the positive influence of supplemental pollen on immune function. However, pollen feeding can stimulate colony growth and this can improve colony survival.

**Electronic supplementary material:**

The online version of this article (10.1007/s10493-020-00562-7) contains supplementary material, which is available to authorized users.

## Introduction

Parasitic mites are a common vehicle for pathogen transmission in plants and animals enabling diseases to spread to new individuals and persist in host communities (see examples in Combes 2005). A parasitic mite that transmits pathogenic viruses in honey bee colonies is *Varroa destructor* Anderson and Trueman. Varroa is an ectoparasite of larval and adult honey bees that transmits and activates several single-stranded RNA viruses (Acute bee paralysis virus, ABPV; Black queen cell virus, BQCV; Israeli acute paralysis virus, IAPV; Kashmir bee virus, KBV, Sacbrood bee virus, SBV and Deformed wing virus, DWV) (Chen and Siede 2007; Runckel et al. 2011; Grozinger and Flenniken 2019). The combination of varroa parasitism and virus infection has been devastating to honey bees and beekeepers as varroa is now the primary cause of colony losses worldwide (Genersch et al. 2010; Guzman-Novoa et al. 2010; van Dooremalen et al. 2012; Martin et al. 2012).

The parasitism cycle for varroa begins when mature female mites (called mother mites or foundresses) enter brood cells just before they are sealed (reviewed in Rosenkranz et al. 2010). Under the sealed cell, the foundress lays an egg that develops into a male. Subsequent eggs develop into female mites that mate with the male. The foundress and her offspring feed on the larva and pupa, and transmit viruses. When the adult bee emerges from the capped cell, the foundress mite and her daughters (average of 1.5 in worker cells and 2.7 in drone cells) leave the cell and attach to adult bees as ‘phoretic mites’. Most commonly, phoretic mites attach to young worker bees that care for developing brood (i.e., nurse bees). Mites distinguish nurse bees from older workers by the differences in their cuticular hydrocarbon profiles (Del Piccolo et al. 2010; Cervo et al. 2014). Adult bees parasitized during development have suppressed immunity, increased susceptibility to various pathogens (Nazzi et al. 2012; Yang and Cox-Foster 2005), and reduced longevity (Kovac and Crailsheim 1988; DeJong and DeJong 1983).

Colonies that are heavily infested with varroa eventually collapse from the combination of parasitism and disease (Genersch et al. 2010; Guzman-Novoa et al. 2010; van Dooremalen et al. 2012). However, it should take more than a year for a colony established with low mite numbers to become heavily infested because the reproductive rate of varroa is relatively low (DeGrandi-Hoffman and Curry 2004). Often though, mite populations in the late fall are larger than expected from mite reproduction alone even if miticides are applied in late summer (Le Conte et al. 2010; DeGrandi-Hoffman et al. 2014). There is evidence that the rapid population increase especially in the fall is from mites migrating into colonies on foragers. Mites can attach to foragers that are robbing weak colonies collapsing from high mite infestations (Sakofski et al. 1990; Frey et al. 2011). Mites also may attach to foragers in their own colonies that then drift to other hives. In the summer, the proportion of foragers with mites (FWM) is low, but the frequencies increase in the fall (Frey and Rosenkranz 2014). Over time the movement of varroa among colonies may significantly contribute to the growth of the mite population (DeGrandi-Hoffman et al. 2014, 2016).

Though varroa numbers may increase through reproduction and migration, feeding colonies pollen might keep virus levels low due to the positive effects of pollen on immunity (Alaux et al. 2010; Di Pasquale et al. 2013; DeGrandi-Hoffman and Chen 2015; Dolezal et al. 2019). Specifically, pollen rich diets may mitigate the negative effects that DWV have on immune signaling and the melanization response (Di Prisco et al. 2016), though it may not reverse them (Alaux et al. 2011). Recent evidence indicates that while good nutrition may not reduce virus levels, it can improve bees’ tolerance and resilience to viral infections (Dolezal et al. 2019), and improve the longevity of adult workers parasitized by varroa as pupae (Annoscia et al. 2017).

An unknown factor in the nutrition-varroa-virus relationship is the contribution that FWM have on virus levels. A plausible scenario is that mite population growth is correlated with the frequency of FWM (DeGrandi-Hoffman et al. 2016), and virus levels rise with mite populations (Francis et al. 2013), so virus levels might be related to the frequency of FWM. To test this scenario, we established colonies and measured the frequency of FWM, mite population sizes and virus levels. Because pollen feeding can improve honey bee immune function and tolerance to viral infections, we established colonies with and without supplemental pollen feeding to determine nutritional effects on virus levels. To evaluate if colonies with supplemental pollen feeding differed in nutritional state from unfed, we compared adult and brood populations and expression levels of the nutritional marker gene, *vitellogenin* (*vg*) between fed and unfed colonies. *Vg* is the major honey bee storage lipoprotein and has the greatest expression in workers nursing brood (5–15 days of age) (Fluri et al. 1982; Crailsheim et al. 1992). If supplemental pollen feeding increased nutrient availability, fed colonies would be larger with more brood and higher *vg* expression levels in the fat body of nurse bees than unfed colonies (Fluri et al. 1982; Amdam et al. 2003). If varroa numbers increase in fed and unfed colonies but virus levels are lower in fed colonies, pollen may provide a level of resistance by improving immune response. If virus levels are similar between colonies with and without supplemental pollen, but more of the fed colonies survive, than pollen feeding might enable colonies to better tolerate stress from varroa and the viruses they transmit.

## Materials and methods

### Overview of experiment

Thirty-two colonies were established in April of 2017 with 3 lb (1.35 kg) packages of bees from Koehnen and Sons (Ord Bend, CA, USA) and headed with marked Italian queens (*Apis mellifera ligustica*). Colonies were equally divided between two locations: the University of Arizona West Agriculture Facility, Tucson, AZ, USA (site 1) and the Carl Hayden Bee Research Center, Tucson, AZ, USA (site 2). The sites were 4.8 km apart. These areas are in mid-altitude desert climates (latitude and longitude: N32_1301800, W110_5503200) where temperatures are conducive for honey bee flight until November (average November min and max temperature: 8 and 23 °C). All colonies initially contained approximately 9000 adult bees and a laying queen. There were managed colonies in nearby apiaries at both sites. Varroa were controlled in nearby apiaries with commercial miticide treatments (ApiGuard; Vita Bee Health, Basingstoke, UK) applied using manufacturers’ instructions to prevent colony loss. Miticides were not applied to our experimental hives.

At both sites, the 16 colonies were divided into two treatment groups. Eight colonies were fed pollen patty weekly (hereafter referred to as ‘fed’ colony). The patties weighed 454 g. To be certain that fed colonies had pollen ad libitum, we provided 1–2 patties every week (depending on consumption) from July 1 through December 15. Each week the fed colonies completely consumed the pollen patties we provided. Eight colonies at each site were not fed (unfed) except for a 1-week period (September 15–22) when they began consuming their brood, and we were concerned about losing the colonies to starvation. At this time, the unfed colonies were provided with 114 g of pollen patty. No additional pollen was provided to the unfed treatment. Pollen patties were made of 33.3% polyfloral corbicular pollen purchased from Natural Foods (Toledo, OH, USA), 33.3% granulated sugar and 33.3% Drivert sugar (C&H Bakers Drivert Pure Cane Sugar; Domino Foods, Yonkers, NY, USA).

### Colony measurements

Combs with bees and brood and stored pollen were measured monthly in all hives at both sites from July until December. Colony measurements were made on Langstroth deep frames (comb dimensions: 48.3 × 2.7 × 23.2 cm). Brood (sealed and unsealed), bees, and stored pollen were measured on both sides of each comb using a grid with 5 × 5 cm squares that covered the entire side of a comb (DeGrandi-Hoffman et al. 2008; Delaplane et al. 2013). The grid was placed above each side of a comb, and the number of squares with bees, brood or stored pollen was counted. Estimates of areas covered by bees, brood and pollen were summed for all combs, and used to estimate total combs with bees and brood and the size of adult bee and brood populations using methods described in DeGrandi-Hoffman et al. (2014). A colony was classified as ‘dead’ if it had less than four combs of bees in December since colonies of this size or smaller cannot thermoregulate the winter cluster, rear enough brood for the colony to grow, or defend the colony from being robbed by larger colonies in the apiary. Final observations of overwinter colony survival were made in March.

### Estimating varroa populations in colonies

Populations of phoretic mites and the proportion of purple-eyed pupae that were infested with mites were estimated monthly in all hives from July until December. Phoretic varroa populations were estimated in each colony using the ‘sugar shake’ (e.g., icing sugar) method described in (Dietemann et al. 2013). We brushed approximately 300 bees from comb with open brood into a glass jar with wire screen lids. Powdered sugar (0.5–1.0 tablespoons) was added through the wire screen lid, and the jar was gently rolled to cover the bees with sugar. The jar was set aside in the shade for 2–3 min, and then inverted and shaken vigorously over a white aluminum pan containing 2.5 cm of water. The mites were counted in the pan. A subsample of the bees from the jar (30 bees), were placed in liquid nitrogen for later virus analysis (10 bees) and *vg* expression (20 bees). The remaining bees were returned to their colony. The mite counts were converted to ‘mites per 100 bees’.

The proportion of worker cells with purple-eyed pupae (within 24 h of emergence) that contained a foundress mite (i.e., fully pigmented adult female) was estimated by examining 80–100 cells per colony. Cells with mites were classified as ‘infested cells’. Subsamples of the pupae (n = 4 per colony) were placed in liquid nitrogen for later virus analysis. There were not enough drone cells in the colonies to generate meaningful data, so these were not sampled.

We estimated the total number of phoretic mites in a colony using measurements of mites per 100 bees and combs of adult bees taken during each sampling period by: combs of adult bees * 2506 workers per comb * mites per 100 bees. Similarly, an estimate of total mites in sealed cells was obtained by: 5200 cells per comb * combs of sealed brood * proportion of sealed cells with mites (DeGrandi-Hoffman et al. 2014).

### Measuring the population of FWM

FWM were collected from colonies using techniques previously described in DeGrandi-Hoffman et al. (2016). Colony entrances were modified so that foragers exited and entered the hive through a 4.76-cm diameter PVC tube. The tube was 61 cm long, with a slit cut across the midpoint. During each sampling interval, a screen was dropped into the slit within the tube to separate foragers entering (incoming) and leaving (outgoing) the hive. Foragers from all colonies at both sites were sampled weekly from July 19 to November 29 when conditions were conducive for foraging. Samples of incoming and outgoing foragers were shaken into separate jars and immediately placed in a cooler containing wet ice. The samples were refrigerated until the foragers became immobile to facilitate examination for phoretic mites. Bees that had a mite attached to its body were counted as FWM, and those that did not have a mite attached were ‘foragers without mites’. A subsample of foragers with and without mites (n = 4 foragers per colony) was placed in liquid nitrogen for virus analysis.

### Evaluating colony nutritional state by *Vg* expression in fat body

Workers collected on combs with uncapped brood during sugar shake sampling for varroa were assumed to be of nurse bee age. The fat bodies from the workers (n = 10 per colony) were dissected by pinning each bee on a wax plate exposing the dorsal surface. A lateral incision was made and the cuticle was pinned to the side to expose the internal structure of the abdomen. The digestive tract, sting apparatus, and ovary tissue were removed and all available fat body was sampled using forceps. Samples were pooled by colony and sampling date into a bead beating tube containing 0.55 mm diameter silica beads placed on dry ice. After homogenizing the tissue in Qiagen buffer RLT/β-mercaptoethanol using a bead beater, RNA was extracted using the RNeasy Mini Kit (Qiagen, Germantown, MD, USA). The resulting RNA sample was treated with DNase (Ambion; ThermoFisher Scientific, Waltham, MA, USA) to digest any remaining genomic DNA. Complete DNA digestion was confirmed with a PCR on the RNA using *actin* primers that yielded no product. The DNA-free RNA was subjected to a cDNA synthesis reaction (RevertAid; ThermoFisher).

The resulting cDNA was used in quantitative PCR reactions to assay the expression of *vg* relative to the housekeeping gene *actin*. The *vg* and *actin* primers were used previously by Corby-Harris et al. (2014). We performed each reaction in triplicate using the SYBR-based SsoAdvanced Universal SYBR Green Supermix (BioRad, Hercule, CA, USA) according to the manufacturer’s instructions. Cq values were averaged across the three technical replicates, and the expression estimates were obtained using the 2^−ΔΔCt^ method (Livak and Schmittgen 2001).

### Virus analysis

Virus levels were determined in purple-eyed pupae and nurse bees collected from sugar shake samples by pooling samples of four individuals per colony. Samples of foragers with and without mites were analyzed as individual bees collected from each colony. Samples of adult bees or pupae were ground with a pestle in a 1.5-ml Eppendorf tube, and homogenized in TRIzol regent (Invitrogen—Thermo Fisher Scientific) for total RNA extraction following the manufacturer’s instructions. The resultant RNA pellet was dissolved in DNase/RNase-Free water containing Ribonuclease Inhibitor (Invitrogen). RNA concentration and purity were evaluated using a Nano-Drop 8000 spectrophotometer (Thermo Fisher Scientific). All RNA samples were stored at − 80 °C for later use.

Quantitative real-time reverse transcriptase-PCR (qRT-PCR) was carried out using a CFX384 Touch real-time PCR system (Bio-Rad) with SYBR green as the fluorophore. The validation of reference genes including β-actin, glyceraldehyde 3-phosphate dehydrogenase (GRADH), and ribosomal protein 18 (rpS18) indicated that β-actin had approximately equal amplifying efficiency with the viruses we tested for, i.e., Acute bee paralysis virus (ABPV), Black queen cell virus (BQCV), Chronic bee paralysis virus (CBPV), Deformed wing virus (DWV), Israeli acute paralysis virus (IAPV), Kashmir bee virus (KBV) and Sacbrood virus (SBV). As a result, only β-actin was used for the normalization of qPCR results in the report. The information regarding primer sequences and the size of PCR products were reported previously (Li et al. 2019). The qRT-PCR amplification was carried out in 25 µl reaction mixture consisting of 2 × Brilliant SYBR green qRT-PCR 1-step Master Mix (Agilent, Santa Clara, CA, USA), forward and reverse primers (20 uM each), and RT/RNase block enzyme mixture. The PCR conditions were as follows: 50 °C for 30 min, 95 °C for 10 min, followed by 40 cycles of 95 °C for 30 s, 59 or 55 °C for 60 s, and 72 °C for 60 s, followed with a final extension at 72 °C for 10 min. Dissociation melt-curves were also included at the end of each run to confirm the specificity of primers. The qRT-PCR reaction was replicated 3 × for each sample. The concentrations of viruses were determine by the 2^−ΔΔCt^ method and expressed as fold differences (Chen et al. 2005).

### Statistical analysis

All analyses were conducted using JMP (Cary, NC, USA) or Minitab (State College, PA, USA) statistical software. The effects of supplemental pollen feeding and colony location (site) on adult population size, brood area, stored pollen, mites per 100 bees, proportion of varroa infested pupal cells, and number of virus types detected in nurse bees were evaluated using repeated measures analysis of variance. Because colony populations differed between fed and unfed colonies, we compared phoretic mite populations per colony between treatments after converting estimates of mites per 100 bees from sugar shake samples to total mites per colony (combs of bees * 2506 bees per comb * mites/100 bees) (DeGrandi-Hoffman et al. 2014). The amount of sealed brood also differed between fed and unfed colonies. We estimated the total infested cells per colony using the proportion of sealed brood cells with mites * combs of sealed brood * 4160 brood cells per comb (DeGrandi-Hoffman et al. 2014). Total mites per colony and infested brood cells were compared between sites and between fed and unfed colonies using repeated measures analysis of variance.

Kaplan Meier analysis followed by a log-rank test was used to compare survival between fed and unfed colonies. A colony was classified as ‘dead’ if it had fewer than four combs of bees in December. Friedmann tests were used to determine the effects of feeding on monthly *vg* levels expressed as normalized ΔC_t_ values (Chen et al. 2005). A separate analysis was conducted for each site. Virus levels in purple-eyed pupae, bees collected from sugar shake samples, and foragers without mites collected from July through November were compared between sites and between fed and unfed colonies using Friedmann tests on normalized ΔC_t_ values. Virus levels between foragers with and without mites were compared between sites and fed and unfed colonies with Kruskal–Wallis tests. We could collect FWM from all colonies only in November, so virus levels were compared for November samples only. The relationships among monthly measurements of colony sizes, proportion of infested cells, average proportion of FWM and mites per 100 bees were determined using multiple regression.

The relationships among colony size, mite populations (phoretic and in pupal cells), FWM and virus levels in sugar shake bees and pupae were determined with an Akaike Information Criterion (AIC) guided path analysis d-separation test (Shipley 2013) executed in R with the *piecewiseSEM* package (Lefcheck 2016). Multiple path configurations were proposed and then compiled into a model using a set of multiple linear equations. Each regression represented the directed dependency of two or more observed variables to each other. The top representative models were then selected for fit with the AIC, and pruned so that only the significant explanatory variables were included. With these adjusted models, a final evaluation was performed and the best fitting path configuration was determined.

## Results

### Colony metrics, *vg* expression and survival

**Fig. 1:**
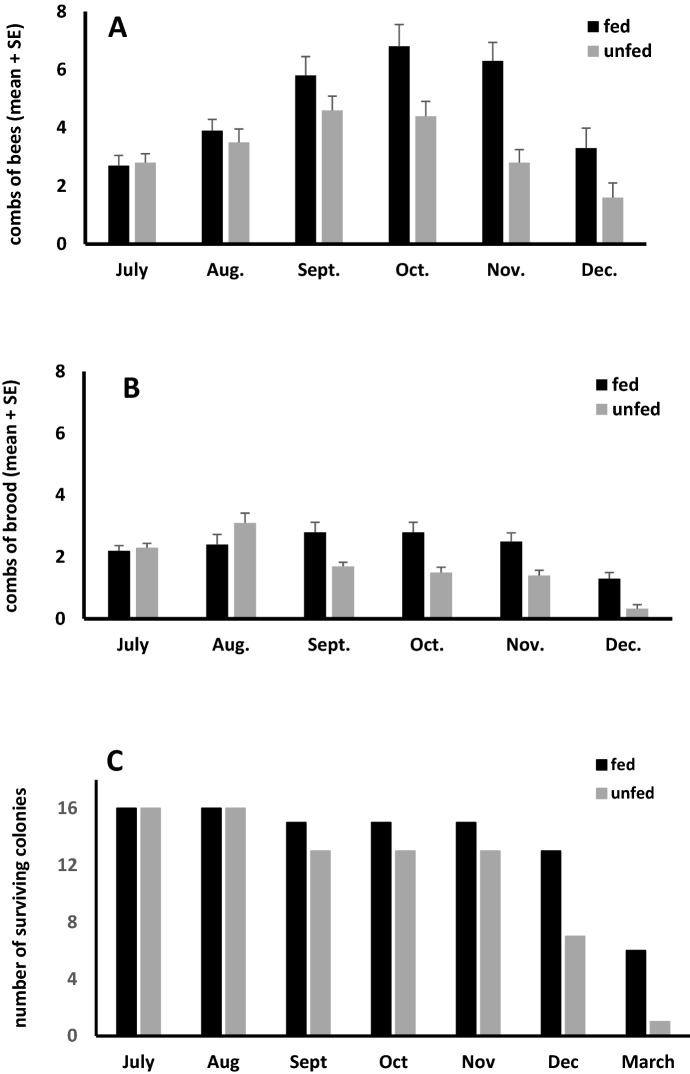
Comparisons of combs of bees (**a**), brood (**b**), and numbers of surviving (**c**) honey bee colonies with (fed) and without (unfed) supplemental pollen feeding. Fed colonies had more bees (F_1,19_ = 5.43, p = 0.03), and brood (F_1,19_ = 19.9, p = 0.0003) and survived significantly longer (χ^2^ = 8.17, df = 1, p = 0.004) than unfed. The study began with 32 colonies (16 fed and 16 unfed)

Colonies at both sites fed pollen patty consumed the entire patty each week. Populations of adult bees did not differ between sites (F_1,19_ = 0.58, p = 0.45), but were larger in fed than unfed colonies (F_1,19_ = 5.43, p = 0.03) (Fig. 1). At both sites, populations in fed colonies peaked in October with about eight combs of bees (20,000 bees). In unfed colonies, populations peaked in September and October with 4.5 combs of bees (about 11,250 bees). By November, populations in unfed colonies dropped, and were about half the size as in October. Fed colonies had a minimal decline from October to November. Brood areas also were similar between sites (F_1,17_ = 0.01, p = 0.90). Fed colonies had larger brood areas than unfed (F_1,17_ = 19.9, p = 0.0003). Brood production did not decline until November in fed colonies, but in unfed colonies, brood rearing began to decline in September. Despite differences in colony sizes, stored pollen did not differ between sites (F_1,16_ = 0.008, p = 0.93) or between fed and unfed colonies (F_1,16_ = 0.61, p = 0.44).

*Vg* expression levels were significantly higher in fed than in unfed colonies at both sites, but were significantly higher only at site 2 (site 1: S_1_ = 1.67, n = 15, p = 0.20; site 2: S_1_ = 5.4, n = 15, p = 0.02). Fed colonies survived significantly longer than unfed (χ^2^ = 8.17, df = 1, p = 0.004) (Fig. 1). Of the 16 colonies established in each treatment group at the start of the study, six of the fed colonies survived the winter and were alive in March compared with one unfed colony.

### Varroa populations

**Fig. 2:**
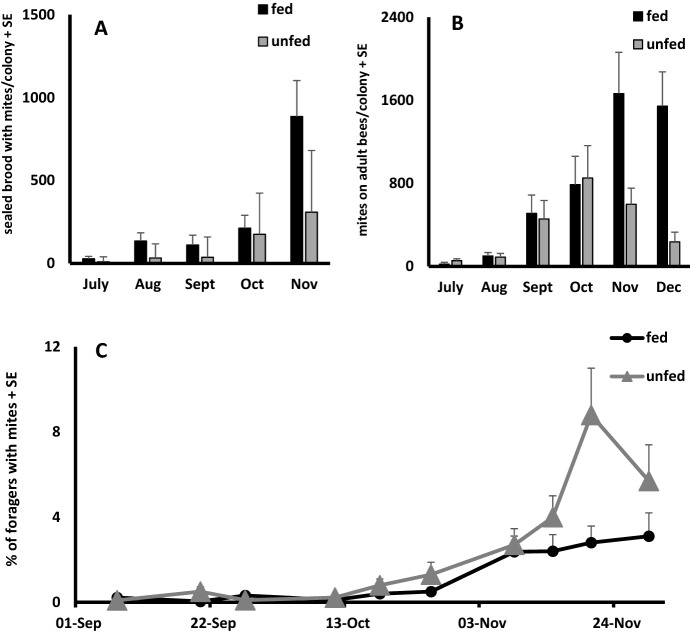
The growth of mite populations on adult bees (**a**) and in sealed brood cells (**b**), and the frequency of capturing foragers with mites at the entrances of colonies (**c**) with (fed) and without (unfed) supplemental pollen feeding. The average number of brood cells infested with mites was higher in fed colonies (F_1,29_ = 6.4, p = 0.017), but mites per colony were similar between fed and unfed colonies (F_1,26_ = 1.01, p = 0.31). Adequate amounts of sealed brood were not available in December (Dec), so only data collected until November are shown. The percentage of foragers with mites was similar between fed and unfed colonies (F_1,33_ = 0.19, p = 0.66)

Comparisons between mite populations in fed and unfed colonies were made in two ways; on the basis of individual bees or cells with purple-eyed pupae and for entire colonies. The number of mites per 100 bees did not differ between locations (F_1,17_ = 0.17, p = 0.69) or between fed or unfed colonies (F_1,17_ = 1.02, p = 0.33). The average number of mites per colony from July to December also did not differ between sites (F_1,26_ = 0.05, p = 0.82) or between fed and unfed colonies (F_1,26_ = 1.01, p = 0.31) (Fig. 2). All fed colonies had ≤ 100 mites (0.20–0.90 mites per 100 bees) in July and August, but this increased to more than 1500 mites in the fed colonies (3–9 mites per 100 bees) by October and November. In the unfed colonies, we estimate < 25 mites per colony in July and August (< 1 mite per 100 bees), and over 800 mites per colony in October and November (7–13 mites per 100 bees).

The proportion of sealed brood cells infested by varroa did not differ between sites (F_1,16_ = 1.56, p = 0.23), or between fed and unfed colonies (F_1,16_ = 0.16, p = 0.69). Similarly, the average number of infested brood cells per colony did not differ between sites (F_1,29_ = 0.81, p = 0.37), but did differ between treatments (F_1,29_ = 6.4, p = 0.02) with fed colonies having more infested cells than unfed. In July, we found less than one mite per 100 cells in fed and unfed colonies (< 30 infested sealed brood cells per colony). By November, fed and unfed colonies averaged 14% cell infestation rates or about 300 (unfed) to 900 (fed) infested sealed brood cells per colony.

We rarely captured FWM at colony entrances in August or September. The frequency of collecting FWM increased throughout October and peaked in late November when 6–9% of the foragers from fed and about 3% from unfed colonies had mites on their bodies. The proportion of FWM did not differ between sites (F_1,33_ = 2.45, p = 0.13), fed and unfed colonies (F_1,33_ = 0.19, p = 0.66), and whether FWM were entering or leaving hives (F_1,33_ = 0.007, p = 0.95).

Multiple regressions were conducted to determine factors affecting the growth of phoretic mite populations (i.e., mites per 100 bees) and numbers of infested sealed brood cells. Growth of phoretic mite populations was significantly correlated with: combs of bees, the proportion of infested brood, and average proportion of FWM captured at the hive entrances (Table 1). The proportion of infested sealed brood cells was correlated with mites per 100 bees and FWM.

**Table 1:** Multiple regression analyses with factors affecting growth of phoretic mite populations, infested sealed brood cells and Deformed wing virus (DWV) levels in honey bee colonies

Response	Predictor	Coefficient	t	p^a^
Mites per 100 bees (R^2^ = 68.4, df = 6, 139)	Constant	− 0.98	1.28	0.20
	Combs of bees	0.55	3.72	< 0.0001^a^
	Combs of brood	0.70	1.50	0.14
	Combs of sealed brood	− 1.62	1.72	0.09
	% of infested pupae	0.53	5.32	< 0.0001^a^
	Mites on pupae	− 0.63	3.03	0.003^a^
	% of foragers with mites	1.29	6.48	< 0.0001^a^
% pupae with mites (R^2^ = 64.9, df = 4, 60)	Constant	0.54	0.22	0.82
	Combs of bees	0.27	0.86	0.39
	Combs of brood	− 0.26	0.55	0.58
	Mites per 100 bees	0.60	4.24	< 0.0001^a^
	Foragers with mites	1.04	3.74	< 0.0001^a^
Levels of DWV—sugar shake bees (R^2^ = 52.8, df = 3, 78)	Constant	17.39	13.92	< 0.0001^a^
	Combs of bees	− 0.83	3.83	< 0.0001^a^
	Combs of brood	− 0.26	2.30	0.024^a^
	Foragers with mites	− 0.83	3.72	< 0.0001^a^

^a^Significant term in the regression equation

### Virus titers

**Fig. 3:**
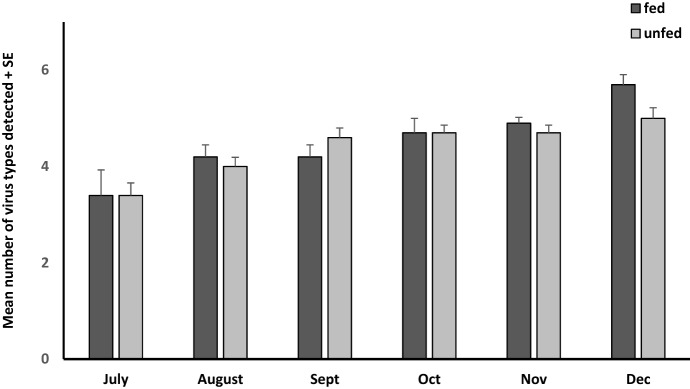
The average number of single-stranded RNA viruses found in nurse honey bees from colonies with (fed) and without (unfed) supplemental pollen feeding. The viruses detected were: Acute bee paralysis virus, Black queen cell virus, Israeli acute paralysis virus, Kashmir bee virus, Sacbrood virus and Deformed wing virus. There was no difference in the number of viruses detected between fed and unfed colonies (F_1,9_ = 0.0056, p = 0.94)

Honey bees typically are attacked by multiple viruses simultaneously (Carrillo-Tripp et al. 2015; de Miranda et al. 2010), and this was the case in our samples. We detected ABPV, BQCV, IAPV, KBV, SBV, and DWV in at least some of the bees during each sampling interval. The number of viruses we detected did not differ between sites (F_1,9_ = 0.16, p = 0.70) or feeding treatment (F_1,9_ = 0.0056, p = 0.94), and increased significantly from July to December (F_5,5_ = 15.8, p = 0.004) (Fig. 3).

Of all the viruses we screened for, only DWV was detected consistently and varied through time in each group we examined. In purple-eyed pupae, DWV levels were similar between sites in unfed colonies, but differed between sites in the fed colonies, with site 2 having lower median levels than site 1 (Table S1). Based on this difference, data were analyzed separately for each site. At both sites, DWV levels in purple-eyed pupae did not differ between fed and unfed colonies. Using July DWV levels as a calibrator, DWV levels in pupae increased by about 100 fold during the study.

**Fig. 4:**
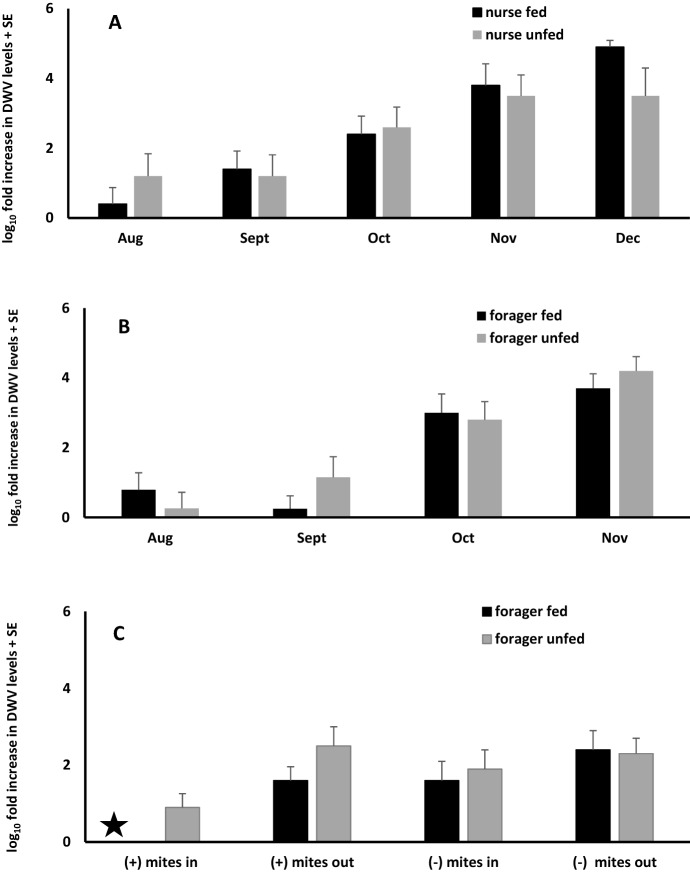
Fold increases in Deformed wing virus (DWV) levels in nurse bees (**a**) and foragers without mites (**b**) from colonies with (fed) or without (unfed) supplemental pollen feeding. Fold differences in DWV levels in foragers with (+) and without (−) mites sampled entering (in) or leaving (out) fed and unfed colonies in November (**c**). July DWV levels (ΔC_t_) were used as the calibrator to estimate fold differences in nurse bees (**a**) and foragers (**b**). DWV levels in foragers with mites captured while entering (mites in) fed colonies, was used as the calibrator (filled star) to compare fold difference between foragers with and without mites in fed and unfed colonies (**c**). Fold increases did not differ between fed and unfed nurse bees (F_1,13_ = 0.077, p = 0.79) or foragers (F_1,30_ = 1.4, p = 0.24). Fold increases in foragers with and without mites also were similar between fed and unfed colonies (F_6,56_ = 1.61, p = 0.16)

DWV levels in bees collected during sugar shakes (i.e., nurse bees) did not differ between sites for fed and unfed colonies (Table S1). Data for both sites were combined, and analyzed to determine if DWV levels differed between fed and unfed colonies. Supplemental pollen feeding did not affect median DWV levels. DWV levels in nurse bees from fed and unfed colonies at both sites showed an increase throughout the study with December values being 10^4^–10^5^ higher than those in July (Fig. 4).

An analysis of foragers (without mites) at site 1 indicated no difference in DWV titers between incoming and outgoing foragers from either fed or unfed colonies (Table S1). Data were combined for incoming and outgoing foragers, and DWV titers were compared between fed and unfed colonies. There was no difference between fed and unfed colonies in median DWV levels. Similarly, at site 2 there was no difference in DWV titers between incoming and outgoing foragers in either fed or unfed colonies. Data were combined (incoming and outgoing foragers), and comparison of fed and unfed colonies showed no difference in DWV levels. A final analysis was conducted comparing DWV levels in foragers between sites. No differences were found in median DWV levels in foragers between sites. Data from both sites were combined to estimate fold differences in DWV in foragers during the study period. DWV levels were 10^4^ higher in November than in July (Fig. 4).

We compared DWV in purple-eyed pupae, nurse bees and foragers to determine if levels differed as bees aged. We found that levels of DWV in nurse bees at sites 1 and 2 were significantly higher than in purple-eyed pupae (site 1: S_1_ = 25.60; site 2: S_1_ = 16.90; both p < 0.0001, n = 40). Comparisons between nurse bees and foragers were made using data from both sites since median DWV levels between sites did not differ. DWV levels in foragers was significantly higher than in the nurse bees (S_1_ = 7.56, p = 0.006, n = 64).

To determine if DWV levels in FWM differed from foragers without mites, we compared DWV levels in November at both sites. Only November data were used because the frequency of capturing FWM from all hives was greatest at this time. At site 1, median DWV levels did not differ between incoming and outgoing FWM in fed or unfed colonies (Table S2). Similarly, foragers without mites (November data only) at site 1 did not differ between incoming and outgoing bees in fed or unfed colonies. Values for incoming and outgoing foragers were combined to test for difference between fed and unfed colonies. DWV levels in FWM from fed and unfed colonies did not differ. DWV levels also did not differ between November samples of incoming and outgoing foragers without mites at site 1 or fed and unfed colonies. Similar trends occurred at site 2 where there was no difference in median DWV levels between November samples of incoming and outgoing foragers without mites in either fed or unfed colonies.

DWV levels in November samples of foragers either with or without mites did not differ between sites, incoming or outgoing foragers, or fed and unfed colonies, so data were combined, and comparisons were made between foragers with and without mites. There was no difference between sites in DWV levels in foragers either with or without mites. Data from both sites were combined to test for differences in DWV levels between foragers with and without mites. In the combined data, median DWV levels were significantly higher in foragers without mites compared with FWM. DWV levels in FWM at both sites were similar to those in nurse bees during November (site 1: H_1_ = 1.7, p = 0.19, n = 32; site 2: H_1_ = 1.9, p = 0.17, n = 20).

A final analysis was conducted to compare fold differences in DWV levels between incoming and outgoing foragers with and without mites. The lowest average virus levels were detected in foragers with mites entering fed colonies, so this value was used as the calibrator to estimate fold differences. The lowest fold increases were in foragers with mites entering fed and unfed colonies. Fold increases in DWV in foragers without mites entering or leaving fed or unfed colonies were about twice as high as FWM entering colonies.

### Path analysis

**Fig. 5:**
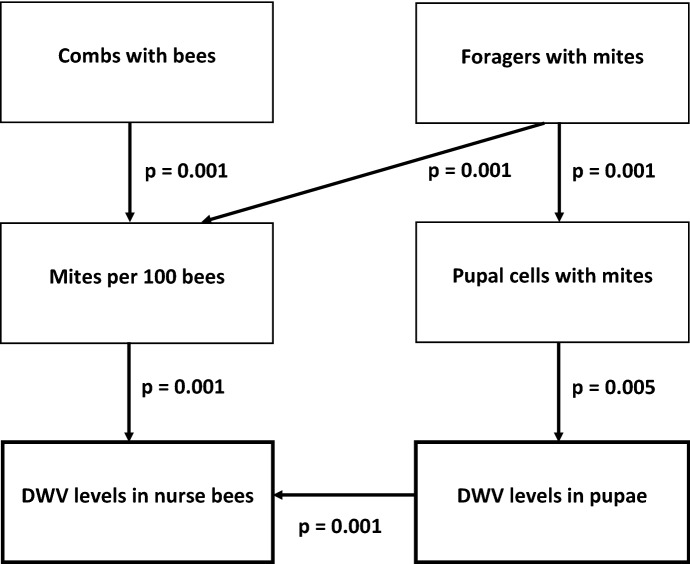
The best fitting path configuration determined by significant explanatory variables selected for fit with the AIC (Fisher’s C = 45.111, df = 16, p < 0.001). Factors significantly affecting Deformed wing virus (DWV) levels in nurse bees and purple-eyed pupae were varroa mites per 100 bees and cells with mites, respectively. DWV levels in nurse bees also were significantly related to DWV levels in pupae. Mites per 100 bees was significantly related to colony size (combs of bees), and the frequency of capturing foragers with mites. Foragers with mites also significantly affected the population of mites in pupal cells

The best-fitting path configuration was determined by significant explanatory variables selected for fit with the AIC (Fisher’s C = 45.111, df = 16, p < 0.001) (Fig. 5). Colony size (combs with bees) and the frequency of capturing FWM significantly affected phoretic mite populations (mites per 100 bees). The proportion of infested pupal cells also was significantly affected by FWM. DWV levels in purple-eyed pupae from uninfested cells were significantly affected by the proportion of cells infested with mites. DWV levels in nurse bees were significantly affected by the size of the phoretic mite population and proportion of cells with mites.

## Discussion

The effects of supplemental pollen feeding on bee populations, *vg* expression, varroa infestations and DWV levels were measured in colonies from July to December. The nutritional states of the colonies differed between treatments. Fed colonies had more bees and brood than unfed colonies and survived longer. Fed colonies also had higher *vg* expression than unfed, though the difference was significant at only one site. Phoretic mite levels were similar between fed and unfed colonies, but fed colonies had more brood cells infested with mites. FWM were captured with equal frequency in fed and unfed colonies, and this may explain the similarity in phoretic mite populations between treatments. The number of viruses detected and the levels of DWV increased between July and December, and were similar between fed and unfed colonies. DWV levels increased as bees aged; pupae had the lowest virus levels followed by nurse bees and then foragers. Interestingly, FWM had DWV levels that were lower than foragers without mites, but similar to those of nurse bees. While pollen feeding positively impacted colony growth, rising numbers of FWM in the fall and subsequent increases in varroa and DWV levels may have minimized the benefits that supplemental pollen had on immune function.

Colonies fed pollen survived longer than unfed colonies even though varroa and virus levels were similar between the treatments. Colonies fed pollen reared more brood and perhaps had workers with greater longevity than those that were not fed. Worker longevity is a fundamental component of colony growth because it affects the size and age structure of the adult worker population and regulates brood rearing and foraging efforts (DeGrandi-Hoffman et al. 1989; Rueppell et al. 2008; Yamada et al. 2019). Annoscia et al. (2017) found that pollen feeding can increase the longevity of workers parasitized during development. In our study, measurements of brood and bee combs were similar between fed and unfed colonies in July and August. However, by September the adult populations that would have been comprised of August brood were lower in the unfed colonies suggesting reduced adult longevity. This trend continued through the remainder of the fall where unfed colonies had 1–1.5 fewer combs of brood, but 2–3 fewer frames of adult bees. Greater worker longevity in the fed colonies in combination with increased brood rearing seemed to extend the survival of colonies stressed by varroa and viruses even when levels rose rapidly in the fall.

Fed colonies had more brood than unfed, and more cells with mites. By November, fed colonies had about 3 × more mites in sealed brood than unfed colonies. The difference in the number of infested cells between fed and unfed colonies, however, did not translate into significantly larger phoretic mite populations. This may be because mite population growth was due only in part to reproduction in brood cells, and was largely the product of migrating FWM especially in the fall (DeGrandi-Hoffman et al. 2016).

Though the benefits of pollen feeding in reducing pathogen levels has been demonstrated in laboratory and field studies (Alaux et al. 2010; DeGrandi-Hoffman et al. 2010, 2016; Di Pasquale et al. 2013; Tritschler et al. 2017; Dolezal et al. 2019), we found that colonies fed pollen did not have lower DWV levels than unfed colonies. There are several possible explanations for our findings. All colonies, fed or unfed, had access to some pollen most of the year. In studies showing an effect of pollen on pathogen load, pollen fed groups were compared with those deprived of pollen and exclusively fed either supplements or sugar syrup. The pollen collected by foragers from unfed colonies, limited as it was during certain times of year, may have been enough to enable immune function to resemble that of fed colonies. Another possible explanation for our results is that we measured effects of supplemental pollen feeding on immune function using virus levels in randomly selected bees that may or may not have had deformed wings. We did not compare the frequency of workers with deformed wings in fed and unfed colonies, which might have revealed differences in immune function that were not apparent in the random samples of nurse bees we collected and analyzed. Future studies should include this measurement as an additional indicator of the effects of pollen feeding on immune function. Another possible reason we did not detect differences in virus levels between fed and unfed colonies is that DWV and varroa levels were low at the start of the study and barely increased from July to October. After October, the frequency of FWM increased in fed and unfed colonies and mite populations rose sharply. Since DWV levels are driven by mite population sizes and these did not differ between treatments, benefits from pollen feeding might not have been sufficient to counteract the negative impacts of rapidly increasing varroa populations and the viruses they transmit (van Dooremalen et al. 2013).

All viruses associated with varroa parasitism were found in our samples; however, only DWV was consistently detected and had levels increase throughout the study. Others have reported that amounts of DWV in colonies and individual bees can increase drastically in a single season (de Miranda and Genersch 2010; Nazzi et al. 2012), and our data support this. Though colonies may be infected with several viruses early in the season, DWV often is the predominant virus detected in the fall. This is because of differences in the quantitative dynamics among the viruses transmitted by varroa. Those dynamics can generate a shifting succession of virus infections that ultimately leaves DWV as the predominant infection (Mondet et al. 2014).

In our study, DWV levels increased as bees aged; the lowest levels were observed in pupae and the highest in foragers without mites. Increasing virus levels as bees aged were expected as pathogens had more time to replicate. Interestingly, DWV levels in FWM were significantly lower than foragers without mites, and similar to those of nurse bees. If DWV levels are used as a proxy for worker age, FWM might be younger workers that are foraging precociously. There is considerable evidence that workers parasitized during development or infected with DWV have reduced response to brood pheromone, less developed hypopharyngeal glands, and forage earlier than uninfected bees (Natsopoulou et al. 2016; Benaets et al. 2017; Zanni et al. 2017, 2018). If FWM are younger workers, it would explain why these foragers are carrying phoretic mites. Additional studies are required though, to confirm that phoretic mites are attracted to precocious foragers that then are identified as FWM.

Colony losses over the winter have not been substantially reduced since the historic levels that occurred in 2006–2007 (vanEngelsdorp et al. 2007). Presently, many colony losses might be due to varroa moving among hives and re-infesting those previously treated for mites in the fall (Frey et al. 2011; Frey and Rosenkranz 2014). Our path analysis indicates that DWV levels increase with mite numbers that are driven by the frequency of FWM. The path configuration is a road map for how colonies can achieve high DWV levels especially in the fall, and perish during the winter (Highfield et al. 2009; Genersch et al. 2010). However, our findings suggest that some colonies might be rescued with good nutrition. The benefits from pollen feeding may not be reduced varroa parasitism or viral infection, but instead greater tolerance to their deleterious effects.

## Electronic supplementary material

Below is the link to the electronic supplementary material.

Supplementary file1 (DOCX 17 kb)

Supplementary file2 (DOCX 18 kb)
