# Supplementary material for: Can supplementary pollen feeding reduce varroa mite and virus levels and improve honey bee colony survival?
Source: Exp Appl Acarol. 2020 Oct 30;82(4):455–73. doi: 10.1007/s10493-020-00562-7 (PMC7686192; doi:10.1007/s10493-020-00562-7)
Supplement: Supplementary file 1 — Supplementary file1 (DOCX 17 kb) [file 10493_2020_562_MOESM1_ESM.docx]

**Table S1.** Results from Freidman analyses of delta Ct values for Deformed Wing Virus in various honey bee lifestages collected in colonies at two apiary sites from July through December. Colonies were either fed supplemental pollen (fed) or had no additional pollen provided beyond what the bees collected (unfed). Pupae were collected in the ‘purple-eyed’ stage of development. Nurse bees were collected randomly by brushing them from brood frames. Foragers were collected either entering (incoming) or leaving (outgoing) their hives.

| Location | Comparison | Sample type | S | n | P |
| --- | --- | --- | --- | --- | --- |
| site 1 vs. site 2  site 1 vs. site 2  site-1  site-2  site 1 vs. site 2  site 1 vs. site 2  both sites  site 1  site 2  both sites | fed  unfed  fed vs unfed  fed vs unfed  fed  unfed  fed vs unfed  fed – incoming vs outgoing  unfed – incoming vs outgoing  fed vs unfed  fed – incoming vs outgoing  unfed – incoming vs outgoing  fed vs unfed  site 1 vs. site 2 | purple-eyed pupae  nurse bees  foragers    all foragers  foragers    all foragers  all foragers | 7.20  3.20  0.00  0.00  0.25  1.67  1.13  0.17  0.17  0.08  1.50  0.67  3.00  0.38 | 20  20  20  20  16  16  32  24  24  24  24  24  24  96 | 0.007*  0.07  1.0  1.0  0.62  0.20  0.29  0.68  0.68  0.77  0.22  0.41  0.08  0.54 |

*significant difference at p <0.05 level
