# Supplementary material for: Can supplementary pollen feeding reduce varroa mite and virus levels and improve honey bee colony survival?
Source: Exp Appl Acarol. 2020 Oct 30;82(4):455–73. doi: 10.1007/s10493-020-00562-7 (PMC7686192; doi:10.1007/s10493-020-00562-7)
Supplement: Supplementary file 2 — Supplementary file2 (DOCX 18 kb) [file 10493_2020_562_MOESM2_ESM.docx]

**Table S2.** Results of Kruskal-Wallis analysis of paired data comparing median delta Ct values for Deformed Wing Virus in foraging honey bees captured at hive entrances at two apiary sites. Two groups of foragers were collected: foragers with *Varroa* mites on their bodies (FWM), and those that were not carrying *Varroa* (foragers without mites). Foragers were collected while entering (incoming) and leaving (outgoing) colonies. Colonies where foragers were collected were either provided with supplemental pollen (fed) or had no added pollen (unfed).

| Location | Sample type | Comparisons  Median Δ Ct values | H | n | P* |
| --- | --- | --- | --- | --- | --- |
| Site1  Site 2  Both sites | fed  unfed  fed  unfed  FWM  foragers without mites  fed  unfed  fed  unfed  FWM  foragers without mites  FWM  foragers without mites  All foragers- site1 and 2 fed and unfed | incoming outgoing  (FWM)  -1.25 -1.01  2.83 4.60  incoming outgoing  (foragers without mites)  -3.30 -3.09  -2.00 -2.00  fed unfed  -1.42 3.67  fed unfed  -3.09 -2.22  incoming outgoing  (FWM)  -2.17 2.76  4.07 -1.66  incoming outgoing  (foragers without mites)  -4.61 -3.23  -2.43 -3.32  fed unfed  0.77 1.43  fed unfed  -3.92 -2.87  site 1 site 2  -0.81 1.43  site 1 site 2  -2.70 -3.27  FWM foragers without  mites  -0.42 -3.08 | 0.04  0.11  0.01  0.18  0.01  0.59  0.16  2.31  0.00  0.20  0.08  3.55  0.05  1.02  9.04 | 20  19  16  13  41  30  12  12  14  14  24  28  66  57  124 | 0.85  0.74  0.92  0.67  0.93  0.44  0.68  0.13  0.95  0.65  0.77  0.06  0.82  0.31  0.003** |

* degrees of freedom = 1 for all pairwise tests.

**significant at the p < 0.05 level
